# Colorectal resection in endometriosis patients: correlation between histopathological findings and postoperative outcome

**DOI:** 10.1186/s40001-021-00484-z

**Published:** 2021-01-23

**Authors:** Peter Tschann, Nikola Vitlarov, Martin Hufschmidt, Daniel Lechner, Paolo N. C. Girotti, Felix Offner, Burghard Abendstein, Ingmar Königsrainer

**Affiliations:** 1 Department of General and Thoracic Surgery, Academic Teaching Hospital Feldkirch, Carinagasse 47, 6800 Feldkirch, Austria; 2 Institute for Pathology, Academic Teaching Hospital Feldkirch, Feldkirch, Austria; 3 Department of Gynaecology, Academic Teaching Hospital Feldkirch, Feldkirch, Austria

**Keywords:** Endometriosis, Bowel involvement, Colorectal resection, Histopathology, Pain level

## Abstract

**Introduction:**

Endometriosis is associated with a high number of chronic pelvic pain and reduced quality of life. Colorectal resections in case of bowel involvement of endometriosis are associated with an unneglectable morbidity in young and healthy patients. There is no linear correlation established between the degree of symptoms and stage of endometriosis. The aim of this study was to correlate the histological findings to preoperative pain scores in colorectal resected patients with endometriosis.

**Methods:**

Twenty-five patients who underwent laparoscopic colorectal resection for endometriosis between 2014 and 2019 were included in this retrospective study. Pain level was assessed preoperatively and postoperatively via phone call in May 2020. Histopathology was correlated to preoperative symptoms and postoperative outcome.

**Results:**

Average follow-up time was 38.68 months (± 19.92). Preoperative VAS-score was 8.32 (± 1.70). We observed a significant reduction of pain level in all patients after surgery (*p* ≤ 0.005). Pain levels were equal regarding the presence of satellite spots and various degrees of infiltration depth. The resection margins were clear in all patients. Postoperative complications occurred in 6 cases (24%) and anastomotic leakage was observed in 3 patients (12%). Average VAS-score at time of follow-up was 1.70 (± 2.54).

**Conclusion:**

Our data demonstrate that adequate colorectal resection leads to reduction of pain and an increase of quality of life irrespective of histopathological findings. An experienced team is necessary to improve intraoperative outcome and to reduce postoperative morbidity in case of complication.

## Introduction

Endometriosis is a chronic gynaecological disease, defined as the presence of endometrial glands and stroma outside the uterine cavity, predominantly in the pelvic compartment, rarely at the diaphragm, pleura, or pericardium [1–3]. It is an estrogen-dependent chronic inflammatory condition which is associated with pelvic pain and infertility, and affects women in their reproductive period [1, 3]. Endometriosis is not a rare condition: it affects 6–10% of women in the reproductive period, 50–60% of women and teenage girls with chronic pelvic pain, and about 50% of women with infertility [3–5]. The economic burden is high because of health care costs and a decrease in productivity of afflicted patients [6]. Affected women lose about 10 h of work weekly [1, 7].

The pathogenesis is still under debate. The most robust evidence is based on the “retrograde menstruation phenomenon” [1, 3, 8]. Endometrial fragments are driven through the fallopian tubes, possibly by uterine contractions which effect a pressure gradient in the tube. Once the endometrial cells reach the peritoneum, they can implant grow and invade other structures [1]. Possible risk factors for this process are early age at menarche, long duration of menstrual flows, as well as molecular and cellular alterations [1, 9].

Endometriosis lesions can be divided into superficial peritoneal implants, ovarian cysts, and deep nodules or plaques, which can individually involve or infiltrate the parametria, Douglas pouch, rectum, bladder, sigmoid colon, or cecum. The rectum and sigmoid colon are the most frequent involved structures, accounting about 90% of intestinal endometriosis cases [2]. Clinical symptoms are depending on the location and extension of endometriosis disease. Rectal or sigmoid endometriosis are often associated with severe progressive symptoms, such as abdominal and pelvic pain, diarrhoea, constipation, haematochezia, and rarely bowel obstruction symptoms [10]. However, most of the patients’ quality of life is restricted by pain, infertility, and repeated operations or long-term medical therapy [11].

Colorectal segmental resection or local bowel wall excision is the usually recommended procedure in case of bowel involvement. Especially, transmural infiltration requires a segmental bowel resection, and these procedures are associated with unneglectable morbidity. Anastomotic leakage remains one of the most threatening complications after colorectal surgery with an incidence up to 20% [12–14]. This is a life-threating complication, even for healthy and young endometriosis patients.

The aim of this study was to evaluate the histopathology of vertical bowel involvement and the outcome after colorectal resection of patients with deep infiltrating endometriosis. Particularly, we wanted to investigate the relationship between histopathological findings and preoperative symptoms.

## Materials and methods

### Study population

388 patients underwent surgery because of endometriosis at the Academic Teaching Hospital in Feldkirch between January 2014 and December 2019. All patients had a typical previous history for endometriosis and were transferred to the certified endometriosis centre of the hospital. Inclusion criteria were colorectal resection for endometriosis. Patients who only underwent diagnostic laparoscopy or bowel wall excisions were excluded. The study was presented to the Ethics Committee of the Province of Vorarlberg (EK-0.04-289).

### Surgical procedure

Twenty-five patients underwent a laparoscopic colorectal resection in case of deep infiltrating endometriosis between January 2014 and December 2019. The indication for bowel resection was posed by the gynaecologist, and the bowel resection was performed by two specialised colorectal surgeons. All procedures were performed laparoscopically in conventional multiport technique using a Pfannenstiel incision or in reduced port technique using an umbilical OCTO™-port for specimen retrieval. The intraoperative resection margins were defined clinically by each surgeon. Complications and surgical outcome were recorded routinely. Severity of complications was graded according to the Clavien–Dindo classification for surgical complications [15].

### Histological evaluation

**Fig. 1:**
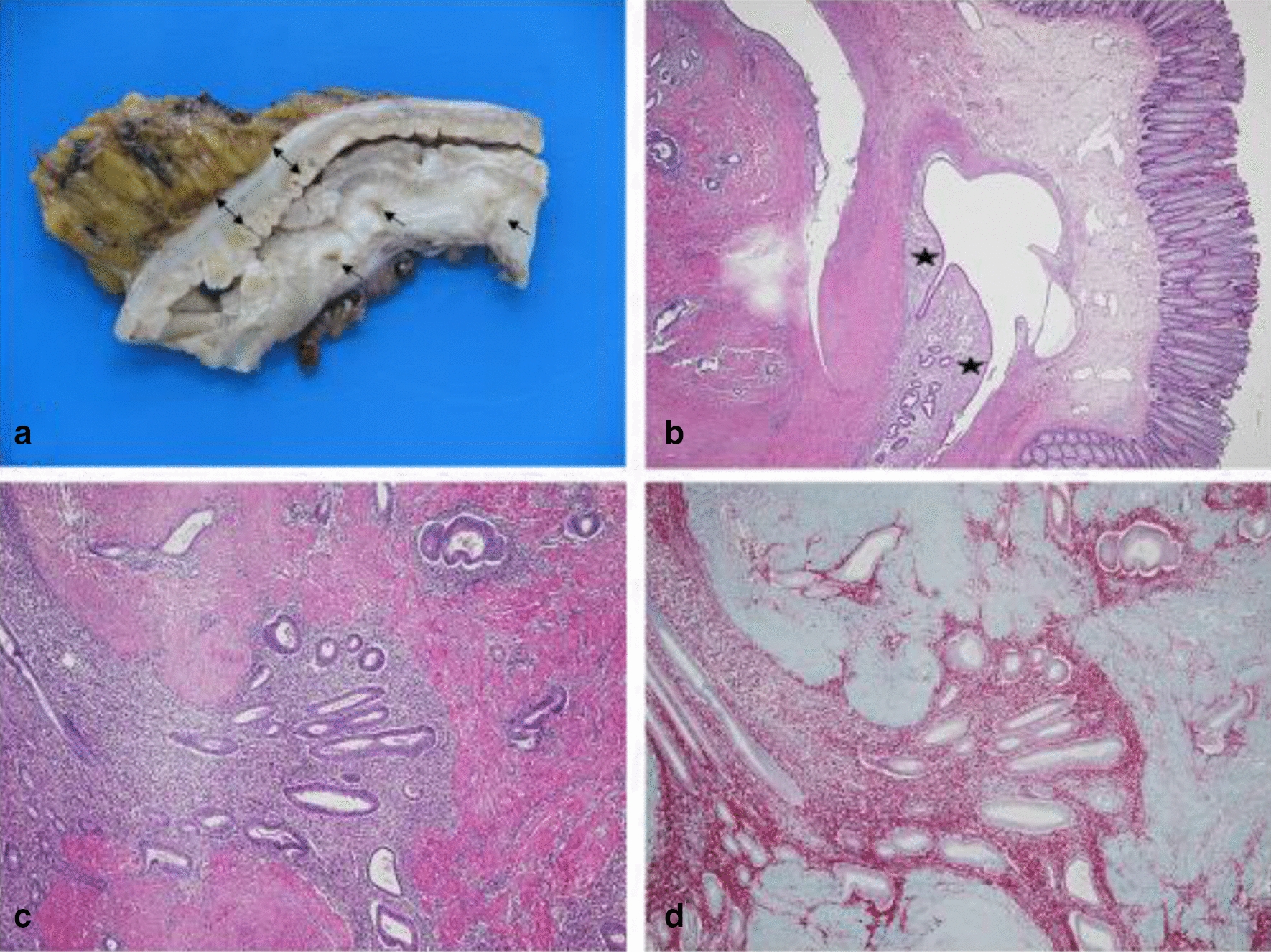
Deep infiltrating endometriosis of the bowel after formalin fixation and paraffin preparation. **a** Endometriotic foci with the white, compact peritoneal tissue (arrow), muscularis propria (double arrow). **b** Microscopy-endometriotic foci infiltrating in the submucosa (star). **c** Microscopy-endometriotic foci in the muscularis propria. **d** Microscopy and Immunohistochemistry. CD10 (+) endometriotic foci in the CD (−) background of muscularis propria

Histopathology and preoperative symptoms were correlated to postoperative outcome. The histopathological examination was performed by our pathologist. The specimen was immediately fixed with 4% formaldehyde. After fixation, a macroscopic description followed by paraffin preparation for microscopical evaluation was done. Vertical involvement and satellite spots (= lesions beside primary endometriosis spot) were recorded as well as the involvement of the resection margins (Fig. 1).

### Preoperative evaluation

Before every procedure, all patients underwent detailed history of symptoms. Preoperative scoring of pain symptoms was performed using a 10-point visual analog scale (VAS) (0 = no pain; 10 = severe pain). In any case of bowel symptoms (pain on defecation, constipation, diarrhoea, bloating, rectal bleeding, and tenesmus), a colonoscopy was performed preoperatively. Magnet resonance imaging was not done routinely.

### Outcome and follow-up

All patients who underwent laparoscopic bowel resection were evaluated by a phone call in May 2020. All data of the interview by call were registered in an electronic format (Microsoft Excel©). Scoring of pain symptoms was performed using the VAS-scale. Patients’ satisfaction and condition were recorded as well as reinterventions because of endometriosis if they were not performed in our hospital.

Continuous data are represented as mean (± SD) and were assessed by the Mann–Whitney *U* test. Data were collected using Microsoft Excel© and analysed with online-based tools. Significance was set at a *p* value of < 0.05.

## Results

Twenty-five patients with an average age of 34.16 (± 5.45) and a mean BMI of 21.76 (± 2.60) underwent laparoscopic bowel resection for deep infiltrating endometriosis. Only 2 patients (12.0%) were scored ASA II and all the others (88.0%) were ASA I. Comorbidities were not observed in our cohort.

Patients’ characteristics, preoperative assessments, symptoms, VAS, and need for pain drugs preoperatively are shown in Table 1.

**Table 1:** Patients’ characteristics, preoperative assessments, symptoms, VAS, and need for pain drugs preoperatively

	*n* = 25
Age	34.16 (± 5.45)
BMI	21.76 (± 2.60)
ASA classification	
I	22 (88.0%)
II	3 (12.0%)
III	0 (0.0%)
Comorbidities	0 (0.0%)
Preoperative assessment:	
Colonoscopy	14 (56.0%)
MRI^a^	19 (76.0%)
Preoperative diagnostic laparoscopy	7 (28.0%)
Preoperative symptoms:	
Tenesmus	2 (8.0%)
Abdominal distension	5 (20.0%)
Constipation	2 (8.0%)
Diarrhoea	3 (12.0%)
Haematochezia	5 (20.0%)
Pain on defecation	6 (24.0%)
Dyspareunia	8 (32.0%)
Dysmenorrhoea	22 (88.0%)
Dysuria	1 (4.0%)
Unfulfilled desire for children	9 (36.0%)
Neuropathic pain	1 (4.0%)
Chronic pelvic pain	4 (16.0%)
Average VAS preoperative	8.32 (± 1.70)
Need for pain drugs during menstruation	22 (88.0%)

^a^MRI, magnet resonance tomography

Preoperative colonoscopy was done in 14 cases (56.0%), MRI was performed in 19 patients (76.0%) with endometriosis symptoms. Diagnostic laparoscopy was done in 7 (28.0%) patients before colorectal resection as a two-step approach. The leading symptom for patient’s presentation to hospital was dysmenorrhoea in 22 (88.0%) cases. Dyspareunia was recorded in 8 (32.0%) and unfulfilled desire for children in 9 (36.0%) patients. Bowel specific symptoms are shown in Table 2. The average VAS preoperatively was 8.32 (± 1.70). 22 (88.0%) patients needed pain killers during menstruation.

13 (52.0%) patients had a history of previous therapeutic laparoscopy for endometriosis, and in 1 (4.0%) patient, a hysterectomy was performed before surgery because of adenomyosis uteri reason.

Perioperative results are shown in Table 2.

**Table 2:** Perioperative results

	*n* = 25
Previous therapeutic laparoscopy for endometriosis	13 (52.0%)
Previous abdominal surgery	14 (56.0%)
Previous hysterectomy	1 (4.0%)
Operation time (min)	200 (± 49)
Operation procedure	
Rectal resection	14 (56.0%)
Sigmoid resection	6 (24.0%)
Ileocaecal resection	1 (4.0%)
Rectal resection + hysterectomy	3 (12.0%)
Rectal resection + ileocaecal resection	1 (4.0%)
Height of anastomosis^a^	9.12 (± 4.46)
Simultaneous gynaecological operation	23 (92%)
Duration of hospital stay (d)	7.37 (± 2.65)
Conversion to laparotomy	0 (0.0%)
Intraoperative complications	0 (0.0%)
Need for ileostomy	4 (16.0%)
Postoperative complications	6 (24.0%)
Anastomotic leakage	3 (12.0%)
Anastomotic bleeding	2 (8.0%)
Infected haematoma	1 (4%)
Dindo–Clavien classification	
IIIa	3 (12.0%)
IIIb	3 (12.0%)
Complication management	
Hartmann procedure	1 (4.0%)
Re-laparoscopy	1 (4.0%
Endoscopic clipping	2 (8.0%)
Endo-VAC therapy	2 (8.0%)
Re-performing surgery	2 (8.0%)

^a^From anal verge in case of rectal or sigmoid resection

**All patients were treated laparoscopically.** The average operation time was 200 min (± 49). A rectal resection was performed in 14 cases (56%), in 3 patients (12%) rectal resection combined with hysterectomy, and in 1 case simultaneous rectal resection with ileocecal resection was performed. Sigmoid resection was done in 6 cases (24.0%). In 1 patient (4.0%), only ileocecal resection was performed. In cases of left-sided colonic resection, the average anastomosis height was 9.12 (± 4.46). In 23 patients (92%), a simultaneous gynaecological resection was necessary because of additional endometriosis spots. A protective stoma was done in 4 cases (16.0%). We observed no intraoperative complications and we had no conversions to laparotomy. The average hospital stay was 7.37 days (± 2.65). Overall, we observed 6 complications (24.0%). In 3 cases (12.0%), anastomotic leakage was diagnosed; anastomotic bleeding was observed in 2 cases (8.0%), and in 1 patient (4.0%), infected haematoma required surgery. Endoscopic clipping was performed in 2 cases of anastomotic bleeding, 2 cases required endo-VAC therapy, and re-laparoscopy was done in case of infected haematoma. A Hartmann’s procedure was necessary in 1 patient.

Pathological findings are shown in Table 3.

**Table 3:** Pathological findings

	*n* = 25
Vertical infiltration	
Mucosa	2 (8.0%)
Submucosa	6 (24.0%)
Muscularis propria	17 (68.0%)
Subserosa	25 (100.0%)
Serosa	25 (100.0%)
Satellite spots	6 (24.0%)
Additional locations	23 (92.0%)
Peritoneum	6 (24.0%)
Vagina	3 (12.0%)
Sacrouterine ligament	7 (28.0%)
Bladder peritoneum	9 (36.0%)
Fallopian tube	3 (12.0%)
Ovary	8 (32%)
Parametries	2 (8.0%)
Clear resection margins	25 (100.0%)

Histopathological assessment showed a mucosal infiltration in 2 patients (8.0%). Submucosal involvement was recorded in 6 cases (24.0%), and muscularis propria in 17 patients (68.0%). All patients had endometriosis infiltration in subserosa or serosa. Satellite spots were observed in 6 cases (24.0%). In 23 patients (92.0%), additional endometriosis locations were resected during the same operation. In all patient’s resection, margins were clear.

Follow-up data are shown in Table 4.

**Table 4:** Follow-up

	*n* = 25
Follow-up time (months)	38.68 (± 19.92)
Reoperation rate for endometriosis	0 (0.0%)
Average VAS-score follow-up	1.70 (± 2.54)
Postoperative symptoms	
Tenesmus	0 (0.0%)
Abdominal distension	0 (0.0%)
Rectal bleeding	0 (0.0%)
Constipation	1 (4.0%)
Diarrhoea	1 (4.0%)
Nausea and vomiting	0 (0.0%)
Pain on defecation	0 (0.0%)
Dyspareunia	0 (0.0%)
Dysmenorrhoea	6 (24.0%)
Dysuria	0 (0.0%)
Chronic pelvic pain	0 (0.0%)
Bladder emptying disorder	1 (4.0%)
Minor LARS^a^	3 (12.0%)
Gravida	5 (20.0%)
Para	2 (8.0%)
*Adjuvant hormonal treatment*	*9 (36.0%)*
Recurrence of endometriosis	0 (0.00%)

^a^LARS, low anterior resection syndrome

During the study period, repeated surgery was not necessary because of endometriosis. The average follow-up time was 38.68 months (± 19.92). The mean VAS-score at time of follow-up was 1.70 (± 2.54). 6 patients (24.0%) reported about mild dysmenorrhoea and intestinal symptoms (constipation and diarrhoea) were rare and only recorded in 2 cases (8.0%). In 1 patient (4.0%), a bladder emptying disorder was observed, and in 3 patients (12.0%), a minor LARS (= low anterior resection syndrome) was observed. After surgery, 5 patients (20.0%) got pregnant, 2 patients (8.0%) gave birth.

Relationship between vertical bowel infiltration and/or additional satellite spots to pre- and postoperative VAS-score at time of follow-up is shown in Table 5.

**Table 5:** Pre- and postoperative VAS-score: correlation between patients with or without satellite spots and vertical bowel infiltration

	Satellite + (*n* = 6)	Satellite−(*n* = 19)		*p* value
VAS-score preoperative	8.66 (± 1.03)	8.21 (± 1.87)		0.97
VAS-score follow-up	1.50 (± 1.97)	1.77 (± 2.75)		0.94

	Mucosa (*n* = 2)	Submucosa (*n* = 6)	Muscularis propria (*n* = 17)	*p* value
VAS-score preoperative	9.00 (± 1.41)	8.50 (± 1.04)	8.17 (± 1,94)	0.93
VAS-score follow-up	0.00 (± 0,00)	1.66 (± 2,42)	1.93 (± 2.74)	0.73

Preoperatively, we observed no difference in pain levels of patients with satellite spots (VAS: 8.66) or without (VAS: 8.21). Moreover, pain level in mucosal infiltration (VAS: 9.00) was similar to patients with submucosal (VAS: 8.50) or muscularis propria infiltration (VAS: 8.17) (*p* = 0.93).

Pre- and postoperative VAS-scores did not differ significantly in case of any postoperative complication. In the group with complications, the average VAS-score preoperatively was 8.66 (± 1.50), 8.21 (± 1.78) in patients without complications (*p* = 0.68). Postoperative VAS was 1.33 (± 2.16) in case of complication and 1.83 (± 2.70) in patients without (*p* = 0.74).

Pain scores preoperatively and at time of follow-up are shown in Table 6.

**Table 6:** Semiquantitative data on pain levels before and at time of follow-up

	VAS-score preoperative	VAS-score follow-up	*p* value
All patients (*n* = 25)	8.32 (± 1.70)	1.70 (± 2.54)	0.00001
Satellite + (*n* = 6)	8.66 (± 1.03)	1.50 (± 1.97)	0.005
Satellite−(*n* = 20)	8.21 (± 1.87)	1.77 (± 2.75)	0.00001
Mucosa (*n* = 2)	9.00 (± 1.41)	0.00 (± 0.00)	0.005
Submucosa (*n* = 6)	8.50 (± 1.04)	1.66 (± 2.42)	0.005
Muscularis propria (*n* = 17)	8.17 (± 1.94)	1.93 (± 2.74)	0.00001

At the time of the follow-up, we observed a significant improvement in pain (VAS: 8.32 vs. 1.70) and in gastrointestinal symptoms (*p* = 0.00001). In case of satellite spots, preoperative VAS was 8.66 (± 1.03), postoperative we found a significant decrease to 1,50 (± 1.97) (*p* = 0.005). Also, in patients without satellite spots, the outcome was significant (8.21 [± 1.87] preoperative vs. 1,77 [± 2.75] postoperative; *p* = 0.00001). Mucosal infiltration showed a preoperative VAS-score up to 9.00 (± 1.41), postoperative we could show a significant reduction to 0.00 (± 0.00) (*p* = 0.005). We observed similar in patients with submucosal involvement (8.50 vs. 1.66; *p* = 0.05) and in case of muscularis propria infiltration (8.17 vs. 1.93; *p* = 0.00001). Patients’ satisfaction after surgery was enhanced. We observed no recurrent disease during time of the follow-up. All patients with left-sided uncomplicated stapled anastomosis routinely were checked with flexible sigmoidoscopy 3 months after the operation. A stenosis or other anastomotic pathologies were not detected. 36% (9/25) of the patients received adjuvant hormonal treatment after the operative procedure.

## Discussion

Colorectal resection in case of bowel involvement of endometriosis is associated with a considerable morbidity in young and healthy patients. Endometriosis is associated with a high number of chronic pelvic pain and reduced quality of life. Bowel involvement causes several intestinal symptoms. Postoperative outcome is related to the removal of involved bowel [16]. In case of deep infiltrating endometriosis with infiltration of several bowel layers, a disc excision or shaving may lead to persistence of bowel symptoms. Several studies demonstrated that laparoscopic bowel resection for deep infiltrating endometriosis is associated with a significant improvement in quality-of-life and pain scores [17–19]. Fedele et al. showed that the risk for recurrence requiring further treatment was significantly higher in patients who did not undergo colorectal resection for endometriosis [20].

Our study reports the result of bowel resection in deep infiltrating endometriosis in correlation to histopathological findings and postoperative outcome. We could show that there is no difference regarding preoperative pain level in correlation to vertical infiltration depth and the presence of satellite spots. Overall postoperative pain level was satisfying and significantly better than preoperative regardless of vertical infiltration or the presence of satellite spots. Moreover, we observed no recurrence and no reoperation for endometriosis at the time of the follow-up.

In most of the cases, the indication for surgery is pain. 88% of our patients need analgesics before surgery. Preoperative the average VAS-score was 8.32. Postoperative, the symptoms decreased significantly to an average VAS of 1.70 (*p* ≤ 0.00001).

It is widely accepted that preoperative assessment should include physical examination, transvaginal ultrasound, and a pelvic MRI. Neither sonography nor pelvic MRI has a 100% effectivity in prediction or confirmation of endometriosis, but they are useful tools in preoperative diagnosis with a high sensitivity and specificity [21]. Routine colonoscopy is not recommended in case of suspected deep infiltrating bowel endometriosis [22, 23]. We would recommend endoscopy especially in case of rectal bleeding to rule out chronic inflammatory bowel diseases and malignancies. Routine diagnostic laparoscopy is obsolete because of repeated admission, persistence of symptoms, and the same effectivity as preoperative transvaginal ultrasound or pelvic MRI [24]. Furthermore, a two-step approach should be indicated restrictively because of a low information content especially for rectal- or rectovaginal endometriosis. To open the rectovaginal space only for diagnostic reason implicates the growth of fibrosis, which makes a second procedure more difficult.

The histological examination showed the presence of satellite spots in 24% of the cases. We could show that preoperative pain levels are similar in patients with satellite spots or without (Table 6). Our data are in accordance to the literature: three studies showed a presence of satellite spots in up to 64% of the cases, and also pain level were similar in patients with and without satellite spots [2, 17, 25]. We observed similar pain levels in patients with mucosal or submucosal infiltration. It is well known that deep infiltrating endometriosis with bowel involvement is associated with a high degree of dysmenorrhoea combined with gastrointestinal symptoms and reduced quality of life. The resection margins of the resected specimen were clear in all cases, and we had no need for recurrence surgery due to endometriosis in the follow-up time. A negative resection margin does not have an impact on postoperative symptoms and outcome in the previous literature [2, 26]. Theoretically, the persistence of endometriosis spots may be responsible for a recurrence disease. It depends on surgeons’ experience and interdisciplinary team work to avoid positive margins, even if there is no correlation between positive margin and persistence of symptoms.

We could show a significant improvement of postoperative pain level in all cases. This underlines the importance colorectal resections in case of deep infiltrating endometriosis. Furthermore, 7 of 9 patients with unfulfilled desire of children got pregnant, and 2 patients gave birth. This is beside pain reduction an important effect of adequate surgery in an interdisciplinary team.

Surgeons must consider that colorectal surgery is associated with a high morbidity in case of complication. We observed complications in 6 cases (24%), and in 3 patients (12%), an anastomotic leakage occurred. A correlation between histopathological presence of satellite spots or vertical bowel infiltration and postoperative pain level did not exist. Postoperative pain level was not higher in case of leakage compared to patients without problems.

Especially, anastomotic leakage increases morbidity and mortality, length of stay, and costs [27]. Only in one case, a Hartmann’s procedure was necessary, and in two cases, an Endo-VAC therapy was possible to preserve the anastomosis. In these two cases, a protective loop ileostomy had been performed in primary procedure. The risk for anastomotic leakage differs in the literature between 7 and 30% [28–30]. To reduce the anastomotic leakage rate, we introduced intraoperative flexible endoscopy for air leak testing immediately after stapling of the anastomosis during the study period. It offers three benefits: vision of perfusion, the integrity of stapler lines, and an air leak test with the possibility to precise the localization of an air leak. In case of negative air leak test (with flexible endoscopy), a protective ileostomy is not indicated necessarily, independent of the height of the anastomosis. In case of positive air leakage intraoperative endoscopy, it facilitates the detection of the insufficiency and offers the possibility of immediate closure by additional suturing. If the test is negative after additional suturing, a diverting ileostomy can be avoided. In case of continuous air leak, re-anastomosis should be considered.

However, patients’ intra- and postoperative outcome strongly depends on the interdisciplinary cooperation between general surgeon and gynaecologist. Colorectal surgery is associated with a high morbidity. Colorectal resections because of endometriosis are challenging due to chronic inflammation. That is why, an experienced team of gynaecologist and colorectal surgeon is needed to define the extent of adequate surgery and for safe and precise performance.

Our study showed that the complete resection of the main lesion leads to a good outcome according to postoperative pain levels and patients’ satisfaction. Weak points of the study are the low number of patients and that it was performed retrospectively.

## Conclusion

Deep infiltrating endometriosis is associated with a high number of preoperative pain and reduction of quality of life. Adequate colorectal resection leads to significant pain reduction. A histological association between satellite spots or vertical bowel infiltration to preoperative pain levels was not significant in our study. An experienced interdisciplinary team seems necessary to avoid intraoperative problems and to reduce morbidity regarding postoperative complications.

## Data Availability

The datasets generated during and/or analysed during the current study are available from the corresponding author on reasonable request.
